# A Receptor Tyrosine Kinase Plays Separate Roles in Sensory Integration and Associative Learning in *C. elegans*

**DOI:** 10.1523/ENEURO.0244-18.2019

**Published:** 2019-08-13

**Authors:** Glenn S. Wolfe, Vivian W. Tong, Emily Povse, Daniel M. Merritt, Gregory W. Stegeman, Stephane Flibotte, Derek van der Kooy

**Affiliations:** 1 Institute of Medical Science, University of Toronto, Toronto, Ontario M5S 1A8, Canada; 2 Department of Molecular Genetics, University of Toronto, Toronto, Ontario M5S 1A8, Canada; 3 Department of Zoology, University of British Columbia, Vancouver, British Columbia V6T 1Z4, Canada

**Keywords:** associative learning, *C. elegans*, chemotaxis, PA14, sensory integration

## Abstract

Associative learning and sensory integration are two behavioral processes that involve the sensation and processing of stimuli followed by an altered behavioral response to these stimuli, with learning requiring memory formation and retrieval. We found that the cellular and molecular actions of *scd-2* dissociate sensory integration and associative learning. This was discovered through investigation of a *Caenorhabditis elegans* mutation (*lrn-2* (*mm99*)) affecting both processes. After mapping and sequencing, *lrn-2* was found to be allelic to the gene, *scd-2*. *scd-2*-mediated associative learning and sensory integration operate in separate neurons as separate processes. We also find that memories can form from associations that are processed and stored independently from the integration of stimuli preceding an immediate behavioral decision.

## Significance Statement

We show that the mutation *lrn-2*, a learning mutant derived from a random mutagenesis screen is allelic to *scd-2*, a receptor tyrosine kinase. Differences in the role of *scd-2* provide the first evidence for genetic, cellular, and behavioral dissociations of sensory integration and associative learning in *C. elegans*. We show that *scd-2* uses different genetic and neuronal pathways for its role in sensory integration versus associative learning. Furthermore, this dissociation shows that sensory integration and associative learning are separate phenomena and that memories can form from associations independent of initial sensory integration. This implies memory formation can be separated from real-time sensory perception.

## Introduction

Although *Caenorhabditis elegans* has a relatively small and simple nervous system compared with mammals, it is capable of behavioral processes such as associative learning and sensory integration (Wen et al., 1997; Ishihara et al., 2002; Giles et al., 2006). Within *C. elegans*, these two behavioral processes are seemingly similar. Associative learning involves the pairing of two sensory stimuli, so that one becomes predictive of the other and leads to a persistent modified behavioral response to the individual conditioned cues. Sensory integration involves the processing of multiple sensory stimuli followed by a modified behavioral response in the presence of the sensory stimuli; however, unlike associative learning, this response may not require the retrieval of a memory. If there are further differences between sensory integration and associative learning (other than presence or absence of memory formation), then such differences may help illuminate the mechanisms of learning and memory function, and indeed how associations are formed.

**Figure 1.**
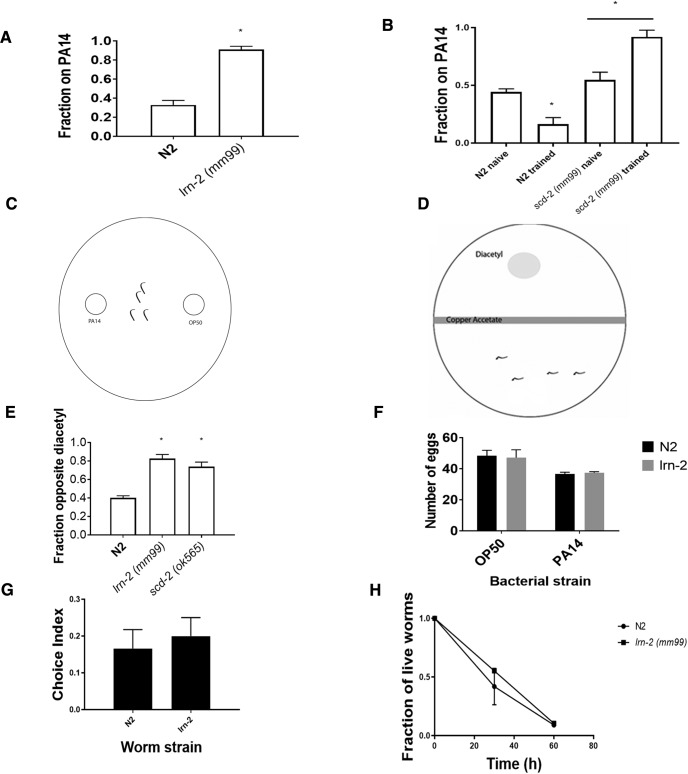
The *lrn-2* mutation displays both associative learning and sensory integration deficits. ***A***, This assay is modified from Zhang et al. (2005, their Fig. 1*A*). It simplifies the procedure by combining the testing and training phases into a single plate. When assayed for associative learning by pairing odor and pathogenicity of PA14, *lrn-2* mutants did not learn to leave the pathogenic lawn, whereas N2 worms made the association and move to the safer OP50 bacteria. Learning was compared with wild-type N2 using a Student’s *t* test. *p < 0.05; *n* ≥ 3 plates; mean ± SEM. ***B***, When assayed for associative learning, using the original method by Zhang et al. (2005, their Fig. 1*A*) that includes separate training and testing trials, *lrn-2* mutants still did not learn a negative association with the pathogenic lawn (one-way ANOVA, *F*_(3,14)_ = 27.54; *p* < 0.0001) with Bonferroni correction (*p < 0.05; *n* ≥ 5 plates; mean ± SEM), whereas N2 worms made the association and chose the safer OP50 bacteria when presented with point sources of OP50 and PA14 and compared with naïve worms that were not exposed to PA14 during training. This is comparable to the modified assay in ***A***. *lrn-2* mutants have an increased attraction to PA14 after training, which may be the result of appetitive learning that is masked by aversive learning in wild-type. Furthermore, this deficit may be caused by an inability to properly detect the pathogenicity of PA14 in the mutant. ***C***, The experimental setup for the testing phase of the PA14 assay. After exposure to PA14 for 4 h, worms are transferred to a new plate, in which they are placed between two point sources of bacteria. One side of the plate has a point of PA14 and the other side has a point of OP50. Worms in the middle can crawl to either side depending one learning. ***D***, The experimental setup for the sensory integration assay, based on Ishihara et al. (2002, their Fig. 1*A*). A Petri dish of NGM with a barrier of copper (II) acetate down the middle and a droplet of diacetyl on one side is used. Worms are placed opposite the diacetyl spot and after 1 h, worms on each side of the copper barrier are counted. Worms that are able to integrate two opposing sensory cues and cross the aversive barrier to reach diacetyl are considered to have normal sensory integration. ***E***, Both *lrn-2 (mm99)* and *scd-2 (sa249)* failed to integrate copper and diacetyl cues, as they crossed the aversive copper barrier to reach an attractive diacetyl odor less than wild-type N2 (one-way ANOVA, *F*_(2,6)_ = 30.83; *p* < 0.001) with Bonferroni correction (*p < 0.05; *n* ≥ 3 plates; mean ± SEM). ***F***, N2 and *lrn-2* both produced similarly fewer eggs on PA14 after 40 h compared with N2. Numbers of eggs produced (both laid and retained in the gonad over the 40 h assay) were analyzed using a two-way ANOVA. There was a significant main effect of bacterial strain (*F*_(1,24)_ = 87.02; *p* < 0.0001; *n* ≥ 6 worms; mean ± SEM), but no significant effect of worm strain nor any significant interaction, showing that N2 and *lrn-2* worms showed similar suppression of egg laying in response to food deprivation. ***G***, Given a choice between PA14 and OP50 *E. coli*, both N2 and *lrn-2* mutants showed a similar naïve preference for PA14 after 1 h (Student’s *t* test; n.s., not significant; *n* ≥ 6 plates; mean ± SEM), despite its pathogenicity. Positive chemotaxis index indicates PA14 approach. ***H***, Both N2 and *lrn-2* worms die at a similar rate when exposed to pathogenic bacteria (PA14), therefore it is unlikely that the difference in learning is caused by a resistance to PA14 pathogenicity. Using two-way ANOVA, there was no significant effect of time or strain.

*lrn-2* (*mm99*) is an EMS (ethyl methanesulfonate, a mutagen that causes random point mutations) derived *C. elegans* mutant with associative learning deficits across multiple associative learning paradigms. Because these deficits include both olfactory and gustatory learning, *lrn-2* is unlikely to be active in either olfactory or gustatory sensory neurons alone. It was originally isolated in a screen for mutants with deficits in associating salt with starvation (Wen et al., 1997), but further research has shown *lrn-2* to have other deficits in associative learning about olfactory cues such as diacetyl (Morrison et al., 1999), and in occasion setting (Law et al., 2004). Here we reveal a deficit in the *lrn-2* mutant in another associative learning paradigm that involves the pairing of the odor and pathogenicity of *Pseudomonas aeruginosa* (PA14) bacteria (Zhang et al., 2005). We have found that *lrn-2* has a deficit in learning about PA14, and the use of this paradigm has led us to a connection between associative learning and sensory integration in *C. elegans* (Fig. 1*A*,*B*). The PA14 learning paradigm used for most of the following experiments is modified from Zhang et al. (2005) by combining the training and testing phases into a simpler assay (Fig. 1*A*), with comparable results to the original method when the original method was employed in our hands (Fig 1*B*,*C*). Although *lrn-2* has an increased approach to PA14 after exposure during training, which could be the result of learning that PA14 has nutritive value, it does not learn that PA14 is pathogenic. This appetitive learning about nutritive value may be masked in the wild-type by aversive learning about pathogenicity. Because *lrn-2* affects multiple associative learning paradigms it is likely that it is active downstream of the sensory neurons. This differentiates it from many of the genes involved in benzaldehyde-starvation associative learning, a *lrn-2* independent memory that apparently is stored in the primary olfactory sensory neuron AWC (Pereira and van der Kooy, 2012). Associative learning using pathogenic bacteria has been shown to require parts of the TGF-β pathway, specifically *dbl-1,* which interacts with *sma-5* in the hypodermis (X. Zhang and Y. Zhang, 2012). However, the genetic and molecular mechanisms that connect pathogenic bacteria associative learning to other paradigms affected by *lrn-2*, such as associative salt learning and diacetyl learning, have not been studied previously.

## Materials and Methods

### Strains

Strains used in this paper include Bristol N2, UT2 *lrn-2* (*mm99*), UT1320 *lrn-2* (*mm99*); *publ-5::GFP,* JT249 *scd-2 (sa249)* V, RB783 *scd-2 (ok565)* V, TY3553 *scd-2 (y386)* V, FX3084 *scd-2 (tm3084)* V, VC980 *fsn-1 (gk429)* III, ZM488 *fsn-1 (hp1)* III, ZM588 *fsn-1(hp1)* III; juls1 [*unc-25p::snb-1::GFP+lin-15(+)*] IV; *scd-2(ok565)* V, *pgcy-28.d::scd-2;pmyo-3::GFP,* UT1321 *pceh-2::scd-2;pmyo-2::mcherry*. Strains were grown at 20°C under standard conditions (Brenner, 1974).

### Sequence analysis and alignment

Sequence reads were mapped to the *C. elegans* reference genome version WS230 (http://www.wormbase.org) using the short-read aligner BWA (Li and Durbin, 2009). Single-nucleotide variants and small insertions and deletions were identified and filtered with the help of the SAMtools toolbox (Li et al., 2009). Each variant was annotated with a custom-made Perl script and gene information downloaded from WormBase.

### 1 h choice assay

PA14 and OP50 were grown overnight in lysogeny broth (LB) medium and resuspended at an absorbance of 1.0 at 600 nm. Twenty-five microliters of each suspension was spotted on opposite sides (4 cm from the center) of a 10 cm Petri dish filled with 30 ml of Nematode Growth Medium and allowed to air-dry for 2 h. 1 μl of 10mm sodium azide was applied to each bacterial spot. Fifty to 150 young adult worms were then placed in the center of the dish and allowed to move freely for 1 h. At 1 h, worms at each bacterial spot were counted.

### Egg-laying assay

This assay was modified from Gardner et al. (2013). Fifty microliters of either OP50 or PA14 were evenly spread on small plates and incubated at 37° for 48 h. Worms were grown from eggs on OP50 for 48 h at 23°C. Two worms were then placed on each plate, which was then sealed and incubated at 20° for 40 h. For counting, adult worms were immersed in a droplet of bleach solution and dissolved. Eggs produced per worm were counted as the number of eggs retained after dissolving and the number of eggs laid on the plate added together.

### Killing assay

<100 young adult worms were placed on small plates containing either OP50 or PA14 lawns on nematode growth medium (NGM). The numbers of live worms were counted at multiple time intervals. Worms that were moving, had a pumping pharynx, and were not sticks were considered to be alive.

### The PA14 associative learning assay

This assay is modified from Zhang et al. (2005, their Fig. 1*A*) to combine training and testing into a single step. Age synchronized young adult worms that were raised on NGM with OP50 at room temperature were used for this assay. Ten centimeter Petri dishes were filled with 30 ml of NGM. Once dry the plates were seeded with two bacterial lawns by pipetting 100 µl of OP50 and PA14 each obtained from overnight cultures grown in LB medium. These lawns were allowed to grow for 48 h before the commencement of the assay. One hundred to 200 worms were washed in M9, and the placed on the PA14 lawn via Pasteur pipette. Once the worms were dry, the plates were sealed in Parafilm and left for the remainder of the assay (4 h). The assay plates were then refrigerated and scored later. The number of worms remaining on the PA14 lawn were counted and divided by the total number of worms on the plate to provide the percentages seen in the assay figures.

In Figure 1*B* the assay included separate training and testing phases. Preparation was the same as above until the plating of worms onto PA14 plates. In this case, training plates contained a PA14 lawn only. One hundred to 200 worms were washed in M9, and the placed on the PA14 lawn via Pasteur pipette. Once the worms were dry, the plates were sealed in Parafilm and left for the remainder of the assay (4 h). Worms were then washed off these plates and placed on the center of testing plates with 50 µl point sources of PA14 and OP50 on either side of the plate for 1 h. In naïve conditions, worms were placed on OP50 plates for 4 h in lieu of PA14 training. These worms were then subjected to the same testing phase as PA14 exposed worms.

### Sensory integration and copper acetate memory experiments

The sensory integration assay was performed as described by Ishihara et al. (2002, their Fig. 1*A*). The assay testing for a learned response to copper (II) acetate is modified from those methods. Ten centimeter NGM Petri dishes used for the training phase were split into three groups. For the paired condition, a barrier of 30 µl of 30 mm copper (II) acetate solution (in water) was spread across the middle of the plate and the gradient was allowed to set for 18 h. Then, age synchronized, young adult worms washed in M9 were placed on one side of the copper barrier, and a 2 µL droplet of 1:100 diacetyl was placed on the opposite side. Worms were dried with a Kimwipe, the plate was sealed with Parafilm and left for 1 h. In the control conditions, worms were either exposed to a 30 µL drop of 30mM copper (II) acetate without diacetyl or a naïve condition with no stimuli added to the plate. The testing phase used plates with a 30 µL drop of 30mM copper (II) acetate left for 18 h. After the completion of the training hour, worms were gently washed off the training plates with M9. Worms from all conditions were then pipetted onto the center of testing plates, dried with Kimwipes, sealed, and left for 1 h. Plates were then refrigerated and scored. Scoring for sensory integration was measured by counting the numbers of worms on each half of the plates and dividing the number on the copper side by the total number on each plate.

### Statistical analyses

Statistical analyses were performed with Prism (GraphPad). The *t* test was used for comparisons of two variables, whereas experiments with multiple conditions were analyzed using ANOVA with Bonferroni corrections for multiple comparisons. For experiments in which groups have two independent variables for comparison, two-way ANOVA was used, with Tukey’s test for multiple comparisons. These analyses are summarized in Table 1.

**Table 1.** Statistical table

	**Data structure**	**Type of test**	**Power (α = 0.05)**
a (Fig. 1*A*)	Normally distributed	Student’s *t* test	0.0005
b (Fig. 1*B*)	Normally distributed	One-way ANOVA	<0.0001
c (Fig. 1*E*)	Normally distributed	One-way ANOVA	0.0007
d (Fig. 1*F*)	Normally distributed	Two-way ANOVA	Bacterial strain <0.0001
			Worm strain 0.8379
e (Fig. 1*G*)	Normally distributed	Student’s *t* test	0.2824
f (Fig. 1*H*)	Normally distributed	One-way ANOVA	0.0003
g (Fig. 1*I*)	Normally distributed	Two-way ANOVA	Strain 0.8493
			Time
h (Fig. 2*A*)	Normally distributed	One-way ANOVA	<0.0001
i (Fig. 2*B*)	Normally distributed	One-way ANOVA	<0.0001
j (Fig. 2*C*)	Normally distributed	One-way ANOVA	<0.0001
k (Fig. 3*A*)	Normally distributed	One-way ANOVA	<0.0001
l (Fig. 3*B*)	Normally distributed	One-way ANOVA	<0.0001
m (Fig. 4*A*)	Normally distributed	One-way ANOVA	0.0034
n (Fig. 4*B*)o (Fig. 4*C*)p (Fig. 4*D*)	Normally distributedNormally distributedNormally distributed	One-way ANOVAOne-way ANOVAOne-way ANOVA	<0.00010.0074<0.0001
q (Fig. 5*A*)	Normally distributed	Two-way ANOVA	Worm strain 0.0217
r (Fig. 5*B*)s (Fig. 5*C*)	Normally distributedNormally distributed	Two-way ANOVATwo-way ANOVA	Training cond. 0.0013Worm strain 0.0986Training cond. 0.6410Worm strain 0.1643Training cond. <0.0001

## Results

### Mapping and identification of the *Lrn-2* mutation locus

Zhang et al. (2005) showed that *C. elegans* forms associative memories about PA14’s olfactory cues and pathogenicity, and the *lrn-2* deficit shown here in this assay (Fig. 1*A*,*B*,*C*) demonstrates that this form of associative learning has shared genetic requirements with salt associative learning, diacetyl associative learning, and sensory integration (Fig. 1*D*,*E*; Wen et al., 1997; Morrison et al., 1999; Ishihara et al., 2002). We first tested whether deficits in PA14 learning might be secondary to the effectiveness of PA14 pathogenicity. However, in both N2 and *lrn-2*, PA14 exposure causes an equivalent decrease in egg production, indicating that both strains are similarly affected by the pathogenicity of PA14 (Fig. 1*F*; Gardner et al., 2013). It is still possible that the learning deficit in *lrn-2* is related to an inability to detect PA14 pathogenicity, despite being affected by it. N2 and *lrn-2* also have equivalent approach to both PA14 and OP50 *Escherichia coli* after 1 h showing that they do not differ in baseline odor sensation (Fig. 1*G*). There is no significant difference in the rate at which N2 and *lrn-2* worms are killed by PA14 pathogenicity (Fig. 1*H*).

**Figure 2.**
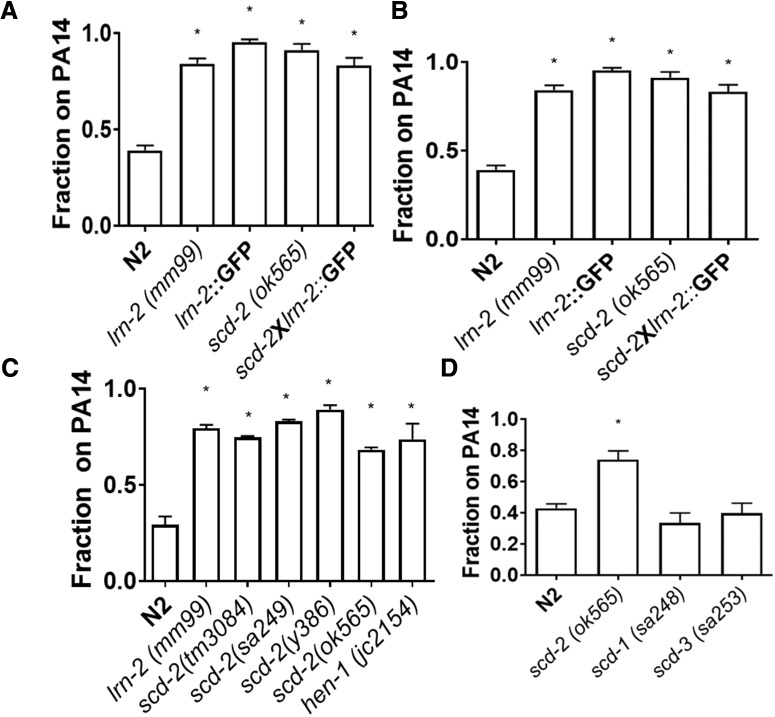
*scd-2* is the locus of the *lrn-2* mutation. ***A***, The F1 progeny of a cross between *scd-2 (RB783)* and a GFP tagged *lrn-2* strain showed that these mutations do not complement, and that the cross progeny still have the mutant PA14 learning deficit compared with wild-type N2 using a one-way ANOVA (*F*_(4,35)_ = 59.50; *p* < 0.0001) with Bonferroni correction (*p < 0.0001; *n* ≥ 7 plates; mean ± SEM). This indicates that the *lrn-2* mutation is likely located in the *scd-2* gene. ***B***, A fosmid containing wild-type *scd-2* was expressed in a *lrn-2 (mm99)* background. When tested for PA14 learning the worms expressing the fosmid and the *pmyo2::mCherry* coinjection marker showed a rescue of N2 like learning. The rescue phenotype was not significantly different when compared with wild-type N2 using a one-way ANOVA (*F*_(4,27)_ = 18.47; *p* < 0.0001) with Bonferroni correction (*p < 0.01; *n* ≥ 4 plates; mean ± SEM). Non-glowing worms were counted as not containing the fosmid and show a learning deficit. These data suggest that expression of *scd-2* can alleviate the mutant deficit, further indicating that it is the locus of the *lrn-2* mutation. ***C***, Four *scd-2* mutant alleles containing both point mutations and deletions replicated the deficit in learning to avoid pathogenic bacteria displayed by *lrn-2*. Learning in *lrn-2* was reduced compared with wild-type N2 worms as shown by a one-way ANOVA (*F*_(5,9)_ = 76.74; *p* < 0.0001) with Bonferroni correction for multiple individual comparisons (*p < 0.01, n.s., not significant; *n* ≥ 3 plates; mean ± SEM). ***D***, Other suppressor of constitutive dauer mutants, *scd-1* and *scd-3* do not show deficits in associative learning about PA14 (one-way ANOVA, *F*_(3,13)_ = 13.18; *p* < 0.001; with Bonferroni correction, *p < 0.01, n.s., not significant; *n* ≥ 3 plates; mean ± SEM). This suggests that the learning deficit is not caused by the dauer-related phenotype alone.

*lrn-2* emerged as a recessive loss-of-function mutation from a random mutagenesis screen (Wen et al., 1997; Morrison et al., 1999). We determined the locus of the *lrn-2* mutation within the genome to investigate further its function within associative learning pathways. Preliminary snip-SNP mapping indicated that a portion of chromosome V was the likely region-of-interest. Whole-genome sequencing was performed on both *lrn-2* and the N2 reference strain, and after analysis of the sequence using SAMTools, a short list of non-silent, non-synonymous mutations was found in the identified region of chromosome V (Li et al., 2009). The only mutant gene that also was not found in the reference strain within the snip-SNP targeted region was *scd-2*. The mutation is a missense mutation causing an A–P amino acid change in the extracellular glycine rich region, which has been shown to be conserved in *Drosophila* and human homologues of *scd-2*, and there is evidence in *Drosophila* that this region is important for protein function (Lorén et al., 2003). In tests to confirm *scd-2* as the site of the *lrn-2* mutation, the *lrn-2* mutant failed to complement an *scd-2* mutant strain, and insertion of a fosmid (WRM0614dF06) containing wild-type *scd-2* into a *lrn-2* mutant background rescued associative learning (Fig 2 *A*,*B*). The complementation test crossed *scd-2* (*ok565*) worms with a glowing *lrn-2* strain. The glowing *lrn-2* strain was created by crossing *publ-*5::GFP worms in a wild-type background into *lrn-2*, selfing the progeny, identifying which progeny displayed the *lrn-2* learning phenotype in a PA14 assay, and confirming with sequencing for the mutation identified previously by whole-genome sequencing. Together, the complementation test and the fosmid rescue confirm that the *lrn-2* mutation is located in the *scd-2* gene.

The *scd-2* gene codes for a receptor tyrosine kinase that is homologous to mammalian anaplastic lymphoma kinase (ALK), which has been shown to affect mouse learning and memory in a suppressive manner (Reiner et al., 2008; Weiss et al., 2012). Worms with mutations in *scd-2* show deficits in sensory integration, and mutations in *hen-1*, the suggested ligand of *scd-2,* are salt learning deficient (Ishihara et al., 2002; Shinkai et al., 2011).

As predicted, three other strains with separate *scd-2* mutant alleles [including a null mutant with a large deletion (*ok565*)] have similar learning deficits to the *lrn-2* strain in the PA14 associative learning assay (Fig. 2*C*). While worms carrying different *scd-2* alleles have learning deficits of slightly varying magnitudes (Fig. 2*C*), this is likely caused by differences in the functional severity of the mutated *scd-2* gene product. Similarly, *lrn-2* mutant worms show a deficit in sensory integration much like *scd-2*(*sa249*), where the deficit is represented as the inability to integrate opposing cues when presented with an attractive diacetyl odor point source, sensed by AWA neurons, beyond an aversive copper acetate barrier, sensed by ASH neurons (Fig. 1*E*; Shinkai et al., 2011). To examine whether the “*scd*” (suppression of constitutive dauer) phenotype had an influence on PA14 associative learning, two other *scd* strains were tested. Neither *scd-1* nor *scd-3* have notable deficits in learning about pathogenic bacteria, indicating that the role of *scd-2* is likely not caused by processes related to a deficit in dauer development (Fig. 2*D*).

### FSN-1 acts upstream of SCD-2 in sensory integration, but not in associative learning

**Figure 3.**
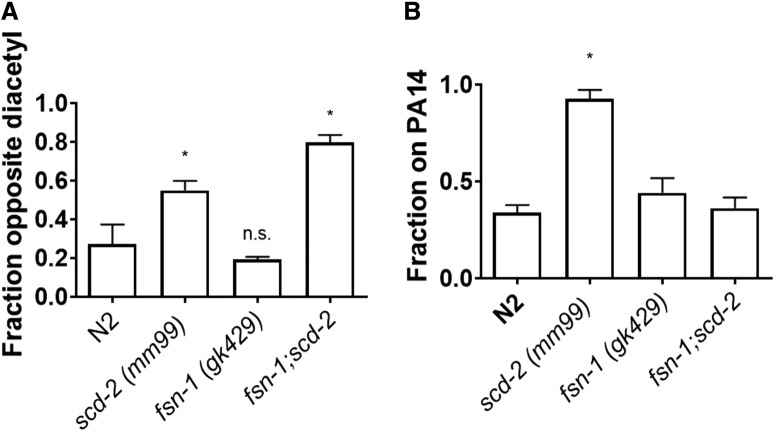
There is a dissociation between associative learning and sensory integration at the genetic level. ***A***, When tested in the diacetyl and copper assay for sensory integration, *fsn-1;scd-2* double mutants showed a deficit similar to *scd-2* single mutants compared with wild-type N2 (one-way ANOVA (*F*_(3,12)_ = 21.29; *p* < 0.0001) with Bonferroni correction (*p < 0.05, n.s., not significant; *n* ≥ 4 plates; mean ± SEM). ***B***, The *fsn-1;scd-2* double mutants did not show a deficit in learning about pathogenic bacteria; instead the double mutants had a similar learned response to N2 or *fsn-1 (hp1)* single mutants (one-way ANOVA, *F*_(5,22)_ = 15.32; *p* < 0.0001) with Bonferroni correction (*p < 0.05, n.s., not significant; *n* ≥ 4 plates; mean ± SEM). This suggests that SCD-2 and FSN-1 have a different interaction in associative learning compared with sensory integration.

The F-Box gene *fsn-1* regulates synapse formation, as part of a SCF ubiquitin ligase complex, and SCD-2 is a downstream target of FSN-1 (Liao et al., 2004; Li et al., 2011). FSN-1 has an intracellular interaction with SCD-2, likely through ubiquitination, causing SCD-2 downregulation and preventing development of abnormal synapse morphology at the neuromuscular junction (Liao et al., 2004). Li et al. (2011) showed that *scd-2* also plays a role in sensory integration and found that FSN-1 suppresses SCD-2’s effect on sensory integration. We also confirmed that the *fsn-1;scd-2* double mutant has a deficit in sensory integration similar to *scd-2* alone (Fig. 3*A*). We predicted that SCD-2 could have a similar relationship to FSN-1 in associative learning. However, the results o*f* testing *fsn-1 (hp1)*, *scd-2 (ok565)*, and double mutants (*fsn-1;scd-2)* in the PA14 associative-learning assay shows that the double mutants demonstrate wild-type learning (Fig. 3*B*). This differs from the double mutant’s sensory integration phenotype (Fig. 3*A*) and indicates that although the *fsn-1* single mutant does not have a deficit, there is an interaction between FSN-1 and SCD-2 that is revealed by the double mutant. This interaction rescues wild-type learning although there is a mutation in *scd-2* present. Thus, the role of FSN-1, as part of the SCF ubiquitination complex, has a different interaction with SCD-2 in associative learning, and this difference in the interaction between these proteins provides evidence for a dissociation of the molecular pathways governing sensory integration and associative learning.

### *scd-2* expression is required in different neurons for sensory integration and associative learning

**Figure 4.**
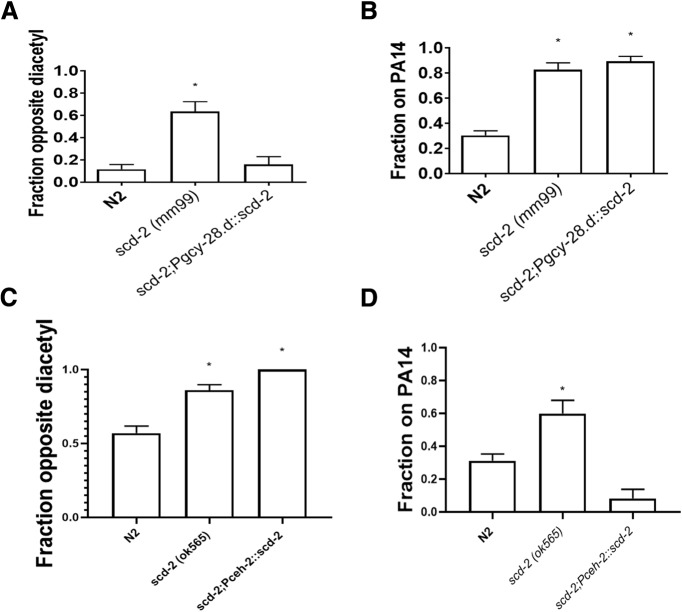
AIA neurons are necessary for sensory integration and NSM neurons are necessary for associative learning. ***A***, *pgcy-28.d::scd-2;pmyo-3::GFP* worms expressed wild-type *scd-2* in AIA interneurons within an *scd-2* mutant background. Expression in AIA rescued the worm’s ability to integrate sensory cues by crossing an aversive copper barrier to reach an attractive odorant. These data replicate the results originally found by Shinkai et al. (2011). Integration was compared with wild-type N2 using a one-way ANOVA (*F*_(2,6)_ = 16.86; *p* < 0.005) with Bonferroni correction (*p < 0.0001; *n* ≥ 8 plates, mean ± SEM. ***B***, Expression of wild-type *scd-2* in AIA did not rescue associative PA14 learning. This indicates that the learning deficit seen in *scd-2* mutants does not require AIA expression, unlike sensory integration (one-way ANOVA, *F*_(2,23)_ = 52.6; *p* < 0.0001) with Bonferroni correction (*p < 0.05, n.s., not significant; *n* ≥ 3 plates; mean± SEM. ***C***, *Pceh-2::scd-2;Pmyo-2::mcherry* worms expressed wild-type *scd-2* in NSM neurons within an *scd-2* mutant background. Expression in NSM did not rescue the ability to integrate sensory cues and cross an aversive copper barrier to reach an attractive diacetyl spot. These results are consistent with the findings that AIA expression is sufficient for sensory integration. Integration was compared with wild-type N2 using a one-way ANOVA (*F*_(3,10)_ = 7.203; *p* < 0.01) with Tukey’s test for multiple comparisons (*p < 0.05; *n* ≥ 4 plates, mean ± SEM). ***D***, Expression of wild-type *scd-2* in NSM neurons within an *scd-2* mutant background rescued associative learning about PA14. This indicates that the role of *scd-2* in PA14 learning requires expression in NSM. Learning was compared with wild-type N2 using one-way ANOVA (*F*_(3,24)_ = 17.17; *p* < 0.0001) with Tukey’s test for multiple comparisons (*p < 0.05; *n* ≥ 7 plates; mean ± SEM).

The site of activity for *scd-2* in sensory integration has been found to be within the AIA interneuron, because expression of wild-type *scd-2* in AIA [under the AIA-specific *gcy-28.d* promoter (*pgcy-28.d::scd-2;pmyo-3::GFP*) within a *scd-2* mutant background] is sufficient to rescue wild-type sensory integration (Fig. 4*A*; Shinkai et al., 2011). If the roles of *scd-2* in sensory integration and associative learning are part of the same process, then it could be expected that AIA interneurons are similarly important for learning, as the mechanism would be shared. However, the differential role of *fsn-1* in mediating sensory integration, as opposed to associative learning, suggests that *scd-2*-mediated learning and memory may require other sites of action. If *scd-2* has independent roles in sensory integration and associative learning, it would not necessarily require AIA expression for learning. Using the same rescue strain as the previous sensory integration rescue experiment, which expresses *scd-2* exclusively in AIA, we tested whether AIA expression was sufficient to rescue associative learning using the PA14 pathogenic bacteria assay (Shinkai et al., 2011). Expression of wild-type *scd-2* in AIA was not sufficient to rescue learning in an *scd-2* mutant background (Fig. 4*B*). This suggests that the site of *scd-2* activity in associative learning and memory formation is in neurons other than AIA. Two remaining candidate neurons with endogenous expression are PVT and NSM (Zhang et al., 2014). NSM neurons are serotonergic and are involved in sensation of bacteria, and control of foraging behaviors, both of which affect PA14 learning (Zhang et al., 2005; Rhoades et al., 2018). Expression of wild-type *scd-2* in NSM using the *ceh-2* promoter in an *scd-2* mutant background was not sufficient to rescue sensory integration (Fig. 4*C*), and indeed sensory integration is AIA mediated. However, expression of wild-type *scd-2* in NSM does lead to a rescue of associative learning when trained to PA14 (Fig. 4*D*), which indicates that wild-type *scd-2* expression in NSM is sufficient for associative learning. Therefore, wild-type *scd-2* must be expressed in different neurons for sensory integration (AIA) and associative learning (NSM), showing that these two phenomena are also dissociated at a cellular level.

### Training to copper acetate and diacetyl produces an *scd-2* independent memory

**Figure 5.**
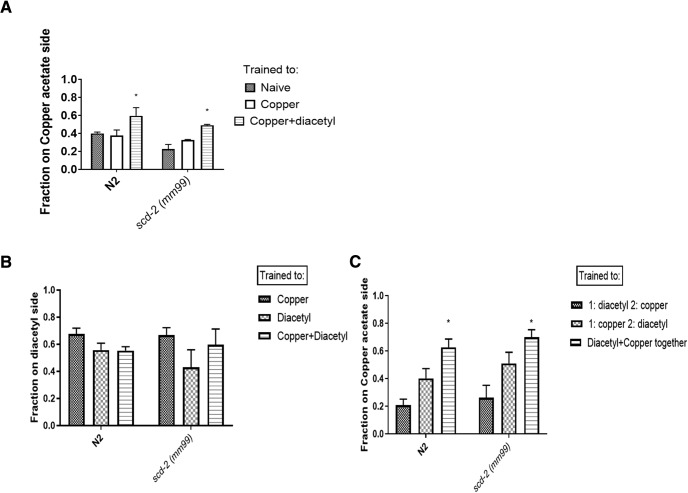
*scd-2* mutants can form an associative memory independent of *scd-2*-mediated sensory integration. ***A***, When N2 and *scd-2* were tested for an associative memory in response to copper acetate post diacetyl and copper training, both strains showed an attenuated aversion to copper acetate. “Fraction on copper acetate side” refers to the fraction of worms on the side of the testing plate that had a point source of copper acetate. This represents the fraction of worms that are attracted to copper acetate. Learning was analyzed using a two-way ANOVA; there was a main effect of worm strain (*F*_(1,12)_ = 6.954; *p* < 0.05), a main effect of training condition (*F*_(2,12)_ = 12.08; *p* < 0.05), but no significant interaction. Tukey’s test for multiple comparisons was performed as a *post hoc* analysis (*p < 0.05 compared with naïve conditions, n.s., not significant; *n* ≥ 3 plates; mean ± SEM). These data indicate that while testing for sensory integration using diacetyl and copper, the worms learn that copper is associated with diacetyl, and thus decrease their aversive response to copper. Because *scd-2* was still able to learn, despite deficits in PA14 associative learning, this memory is formed by a separate mechanism from associative PA14 learning. ***B***, Testing to diacetyl instead of copper does not show learning perhaps because of a ceiling effect. N2 and *scd-2 (mm99)* worms were trained to associate diacetyl and copper, then approach to a point source of diacetyl was measured. However, diacetyl remained highly attractive across control and trained conditions. There is no significant difference between groups. ***C***, N2 and *scd-2 (mm99)* worms were exposed to diacetyl for 1 h, followed by copper for 1 h (and vice versa), and then tested to a copper (II) acetate point source. Copper (II) acetate and diacetyl were also presented together for 1 h in a paired condition following 1 h on a plate with no odorants. These data indicate that diacetyl and copper (II) acetate should be presented simultaneously in order for a learned association to form. Learning was analyzed using a two-way ANOVA; there was a main effect of condition (*F*_(2,41)_ = 19.73; *p* < 0.0001). Tukey’s test for multiple comparisons was performed as a *post hoc* analysis (*p < 0.05 compared with naïve conditions; *n* ≥ 9 plates; mean ± SEM). The slight difference between the two conditions in which copper and diacetyl were presented separately was not found to be statistically significant.

The gene *scd-2* plays roles in both sensory integration and associative learning; roles that may be part of two independent processes that can be dissociated at the genetic and cellular levels. Indeed, sensory integration involves the sensation of two stimuli and an immediate behavioral choice in the presence of both stimuli. This description of sensory integration differs from associative learning only in the lack of the persistence of the behavioral change (in the form of a memory) with associative learning. If associative learning and sensory integration are truly independent processes, an organism should be able to learn while sensory integration is blocked, and vice versa. Furthermore, the same sensory cues that lead to a sensory integration decision could simultaneously form an associative memory. To investigate whether there was a memory formed by presentation of two cues to test for sensory integration, a modified version of the diacetyl-copper sensory integration assay that includes separate training and testing phases was used. This would demonstrate whether diacetyl and copper form an associative memory while the worm is integrating these two cues. During training, N2 and *scd-2* worms were exposed to diacetyl and copper, as in the sensory integration assay (Fig. 1*D*) for 1 h, along with naïve and copper only control groups. Both strains were then tested on plates with a point source of copper acetate for 1 h and their chemotaxis responses were recorded. When trained in presence of both attractive diacetyl and aversive copper acetate, worms form a memory of the association between these stimuli (Fig. 5*A*). This is shown by the attenuation of copper aversion for conditioned worms in the testing phase compared with single stimulus trained animals (Fig. 5*A*). When tested to diacetyl instead of copper, there is no change in diacetyl approach, implying that there is a ceiling effect (Fig. 5*B*). Furthermore, this learned response to copper requires simultaneous presentation of both copper and diacetyl, because there is no learning when presented sequentially (Fig. 5*C*). This indicates that copper approach reflects an associative memory. Because both N2 and *scd-2* worms are able to learn about copper acetate, this memory must be separate from *scd-2*-mediated memories and it must not require *scd-2*-mediated sensory integration to associate copper acetate and diacetyl. Although an *scd-2* mutation often leads to a deficit in learning, *scd-2* worms are able to form a memory when copper and diacetyl are paired. This is possible because there are multiple learning circuits for different types of memories in worms; *scd-2* mutant worms are able to pair benzaldehyde and starvation, and can successfully learn about mechanosensory stimuli (Nuttley et al., 2002). Similarly, not every gene involved in benzaldehyde starvation learning plays a role in *scd-2*-mediated PA14 or salt learning. This is a clear dissociation of sensory integration and associative learning at the level of behavior, suggesting that memories formed by pairing copper acetate and diacetyl are formed independently of the sensory integration of the same two stimuli as measured by the sensory integration assay.

## Discussion

The results of our experiments suggest that wild-type *scd-2* plays independent roles in associative learning and sensory integration. Although the complete genetic and cellular circuits for these two processes are not fully known, they both appear to use some of the same genetic machinery within independent circuits. Associative learning requires a persistent alteration of the behavioral response that can be observed during testing after the initial pairing of cues and in the presence of the conditioned cue alone. This persistent response is caused by the formation of a memory that can be repeatedly accessed when cues are presented at testing. Sensory integration also a change in behavioral response, but this response is an immediate decision after cue exposure, not a persistent learned choice. It is conceivable that sensory integration is a necessary early step in processing sensory information that leads to an associative memory. If this were the case, we would expect to see *scd-2* acting in the same pathways for both processes, but our data suggest that *scd-2* operates in independent genetic and cellular pathways. We describe this difference as a dissociation because it is these data concerning the difference in *scd-2* activity that identify the independence of the genetic and cellular circuits involved.

Associative memories that require *scd-2* cover a variety of sensory modalities (Wen et al., 1997; Ishihara et al., 2002), but remain separate from AWC primary olfactory neuron-mediated associations such as benzaldehyde/starvation pairing (Nuttley et al., 2002), and memories formed during the initial integration of diacetyl and copper acetate. Worms with *scd-2* mutations do not have known deficits in chemosensation, mechanosensation, thermosensation, nor in non-associative learning (Rankin et al., 1990; Morris et al., 1997; Mori, 1999). Further, the present data show that associative memories of a copper and diacetyl pairing can be present in the same *scd-2* mutants that are unable to undergo sensory integration of copper and diacetyl nor PA14 associative learning (Fig. 5*A*, see below). Indeed, the present work provides evidence that sensory integration and associative learning are dissociable at the genetic, cellular, and behavioral levels. These data suggest that there are deeper differences between sensory integration and associative learning in *C. elegans* than simply the formation of separate memories. Not only are sensory integration and associative learning dissociated, but also learned associations can be formed through a process completely independent from sensory integration that must be occurring simultaneously and in parallel. With *scd-2* orthologs present in other species, including mammals (Morris et al., 1997), it is possible that this dual role in dissociated processes is conserved across taxa, providing opportunity to investigate the function of mammalian learning and memory genes in this genetically tractable nematode model.

The transcription factor FSN-1 has an established role in regulating synapse formation at the neuromuscular junction, and *fsn-1* mutants display both abnormal synapse morphology (Liao et al., 2004) and enhanced sensory integration (Li et al., 2011). Experiments with double mutants of *fsn-1* and *scd-2* show an *scd-2*-like sensory integration deficit (Fig. 3*A*; Li et al., 2011). Although this relationship regulates both sensory integration and the neuromuscular junction, the roles of SCD-2 and FSN-1 in associative learning about pathogenic bacteria must be part of an independent pathway because the double mutant does not produce a deficit in PA14 associative learning. Because *fsn-1;scd-2* double mutants are phenotypically similar to *fsn-1* alone in associative learning about pathogenic bacteria, there must be a different interaction between FSN-1 and SCD-2 compared with their activity during sensory integration. If these two proteins did not interact in PA14 learning, the double mutant would be expected to show a deficit similar to *scd-2* alone; the *fsn-1* mutation somehow masks the *scd-2* learning deficit (Fig. 3*A*,*B*). There is further evidence of separate *fsn-1*-dependent and -independent pathways containing shared components from previous research. The insulin-like protein, INS-6, acting through the DAF-2 receptor has an antagonistic relationship with FSN-1 in which reduced insulin signaling rescues the *fsn-1* abnormal synapse morphology phenotype (Hung et al., 2013). Furthermore, INS-6 plays a role in enabling associative learning about pathogenic bacteria by repressing transcription of *ins-7* (Chen et al., 2013). Thus, wild-type *ins-6* plays a role in synapse formation that is phenotypically similar to the role of wild-type *scd-2*, but its role in associative learning may not be through negative regulation of FSN-1. The effect of *ins-6* on learning also happens through a different mechanism than its antagonistic relationship with *fsn-1* in sensory integration; this further supports the notion of that sensory integration and associative learning are independent processes with some shared genetic components. Although a connection between *scd-2* and *ins-*6 remains unknown, we have found in the present results that *scd-2* could have a similar relationship with to *fsn-1* as *ins-6*, in which sensory integration appears to require *fsn-1* suppression but associating pathogens with odor operates independently.

Expression of wild-type *scd-2* from fosmid injection employing the endogenous promotor rescues both sensory integration and associative learning, but the individual cells necessary for each process must be established. We have shown that targeted expression of wild-type *scd-2*, under an AIA interneuron-specific promoter, although sufficient for rescue of sensory integration in an *scd-2* mutant background, is not sufficient for rescue of associative learning (Fig. 4*A*,*B*). However, wild-type *scd-2* expression in NSM did lead to a rescue of the associative learning phenotype in the mutant, so NSM neurons are the cellular location of *scd-2* activity during PA14 associative learning (Fig. 4*D*). NSM neurons endogenously express wild-type *scd-2* (Mckay et al., 2003; Zhang et al., 2014), and are serotonergic, located in the head, play a role in sensation of bacteria, and mediate foraging behavior (Avery et al., 1993; Rhoades et al., 2018). There also is evidence that NSM regulates attractive food related response to bacteria, which could indicate that the present findings concerning the *scd-2* mutation are caused by the inability to properly identify whether PA14 or OP50 are the more attractive or nutritious bacteria (Zhang et al., 2005). These features of NSM are all consistent with the features of PA14 learning, because learning about PA14 requires serotonin, uses bacteria as an unconditioned stimulus, and leads to modified foraging behavior when worms no longer approach PA14. The difference in cellular localization of *scd-2* expression in sensory integration and associative learning provides another dissociation of these two processes at the circuit level. Although *scd-2* is involved in both processes, these data indicate that *scd-2* expression and activity are required in separate neurons in two dissociated circuits that have differing genetic interactions with *fsn-1*. Although neuronal circuits for sensory integration have been proposed previously (Ishihara et al., 2002; Shinkai et al., 2011), the neuronal circuit for PA14 learning is less complete. ADF has been shown to be important for serotonergic activity, *ins-6* and *ins-7* have been shown to communicate through URX and RIA, *dbl-1* activity requires release from AVA to ASI or hypodermal cells (X. Zhang and Y. Zhang, 2012; Chen et al., 2013), and we now know that *scd-2* also plays a role in NSM. Because the wild-type ligand of *scd-2*, *hen-1* can still rescue sensory integration even when expressed in neurons that do not endogenously express it; *hen-1* may not need to be expressed in neurons that synapse directly with NSM for *scd-2* learning (Ishihara et al., 2002). Thus, it is possible that detection of PA14 odors by AWC (Ha et al., 2010) leads to HEN-1 release from AIY, which travels to SCD-2 receptors in NSM, causing an learned alteration of foraging behavior. Nevertheless, how this part of the proposed neuronal circuit connects with the URX, ADF, and AVA neurons will require further study.

The existence of multiple associative learning circuits, both *scd-2* independent and dependent, provides an opportunity to investigate whether sensory integration plays a role in the initial sensory cue processing step in memory formation. *scd-2* Does not have deficits in all forms of associative learning, so the finding that pairing copper acetate and diacetyl leads to the formation of an *scd-2*-independent memory (Fig. 5*A*) is not remarkable on its own. However, the evidence that an *scd-2*-independent memory can be formed after training to copper acetate and diacetyl cues, despite a deficit in integrating those cues, suggests that the formation of such a memory uses a mechanism of sensory association that is independent of the decision-making process during sensory integration. Therefore, sensory integration of opposing stimuli is not the process by which the cues are associated to form memories, and associative learning must be occurring both simultaneously and independently of sensory integration. Although the initial associative mechanism used to form copper acetate and diacetyl associative memories is still unknown, the dissociation at the level of sensory integration and associative learning is consistent with the dissociations of these two behavioral processes at the circuit and molecular levels.

In worms, if associations can be formed independently of sensory integration, then there must be a difference in how a memory is represented versus how integrated stimuli are represented within the worm nervous system, much like the differential processing of contextual cues in mammals (Skinner et al., 1994; Good et al., 1998). One possibility is that sensory integration leads to a blending of two stimuli in a single representation that leads to an appropriate motor response, whereas associative learning encodes an association between the separate representations of two stimuli. For example, integration of copper and diacetyl would create a temporary blended representation that is interpreted as “copper tastes different and better mixed with diacetyl”, and the worm’s motor response would depend on salience of these cues when presented together. Because a sensory integration response is not memory dependent and only functions in the presence of both stimuli, it can be predicted that the worm is responding to a temporary perceived representation of a blended stimulus. Because associative memories can be retrieved after training by a single associated cue without required simultaneous presentation, it is likely that the worm processes associative learning as two separately stored but paired cues instead of a blended representation. Memory recall could activate a representation of the conditioned stimulus that co-activates a separate representation of the unconditioned stimulus and triggers the appropriate behavioral response. Such an engram preserves the associative information learned during training, while allowing post-training retrieval without the presence of both stimuli simultaneously.

Sensory integration and associative learning, although both involve detection and processing of sensory cues followed by a behavioral choice, are dissociated at three distinct levels. Differences in the FSN-1 interaction with SCD-2 dissociate these processes through their molecular mechanisms. Activity of SCD-2 is required in different cells for sensory integration and associative learning, indicating a dissociation of the neuronal circuitry behind these processes. The ability to form *scd-2*-independent associative memories without *scd-2*-mediated sensory integration indicates that these processes are also functionally dissociated at the behavioral level. This dissociation suggests that wild-type *scd-2* is an important regulator of multiple processes. *scd-2* also has been shown to regulate forgetting of memories dependent on AWA sensory neurons, while our findings show an *scd-2*-mediated independent regulation of memory formation of AWC-based PA14 learning (Kitazono et al., 2017) The independent importance of wild-type *scd-2* in forgetting, sensory integration, and associative learning shows that it is an important gene to study for further understanding of nematode behavior. Although the details of the molecular, cellular, and behavioral mechanisms behind sensory integration and associative learning are not fully known, *scd-2* provides evidence that they are two independent behavioral processes governing *C. elegans* behavior. Understanding the nature of such dissociations may be able to inform how learning and memory occur both within the nematode, and potentially, across taxa.

## Synthesis

Reviewing Editor: Lorna Role, NINDS Decisions are customarily a result of the Reviewing Editor and the peer reviewers coming together and discussing their recommendations until a consensus is reached. When revisions are invited, a fact-based synthesis statement explaining their decision and outlining what is needed to prepare a revision will be listed below. The following reviewer(s) agreed to reveal their identity: Catharine Rankin.

This is a re-submission of a manuscript that was previously rejected with re-submission permitted. The reviewers do not ask for any additional experiments, but the manuscript must be completely rewritten as it is fragmented, difficult to read and the conclusions drawn are misleading.

Of primary importance is that the reviewers are in strong agreement that the manuscript, as written, greatly extends the conclusions beyond what are supported by their data. They specifically emphasize that the only conclusions that can be drawn from the current manuscript are that:

1) the alleles they isolated are allelic to scd-2;

2) scd-2 mutants have an altered response to pathogenic bacteria; and

3) putting scd-2 into HSN neurons rescues this altered response to pathogenic bacteria.

The data, in the present form, do not support any other conclusions, much as one may want to argue that they do.

*** As such the authors need to re-write the paper so that it is clearly written and is devoid of the current overreach. This includes changing the title to focus on scd-2, rewriting the body of the paper, rewriting the abstract and significance statement so that these are devoid of over reach and removing the presentation and discussion of the other experiments that are not well supported. ***

Specific aspects of how the manuscript needs to be rewritten are included in the more detailed aspects of the reviews, which are synthesized here:

Overview:

This paper examines the role of lrn-2/scd-2 receptor tyrosine kinase in two processes, sensory integration and associative learning, in the nematode C. elegans. The authors first convincingly present data that lrn-2 mutations were allelic to scd-2 mutations. Scd-2 mutants were previously shown to be unable to integrate the opposing attractive olfactory and repellent gustatory cues, diacetyl and copper sulfate, which are sensed by the AWA and ASH sensory neurons, respectively (Ishihara et al., 2002). The sensory integration response was reported to be dependent on fsn-1 SCF ubiquitin ligase and its target scd-2 (Li et al., 2011; Shinkai et al., 2011).

In repeating these experiments, the authors have obtained different results. In contrast to Li et al. (2011), the authors showed that fsn-1 mutants acted similar to wild type. **Hence, the authors cannot use fsn-1 mutants for epistasis analysis as they report and they need to address the discrepancy in the Results and their interpretations in the Discussion.***

The authors were able to repeat other results (Li et al., 2011; Shinkai et al., 2011) that showed that scd-2 mutants were unable to integrate sensory cues and showed a significantly higher number of animals that did not go towards diacetyl relative to wild type.

In an associative learning assay developed by the Bargmann lab (2005), animals were exposed simultaneously to a nutritious and pathogenic bacterial food source, and subsequently assayed for their bacterial preference; wild-type animals will go towards the nutritious bacteria in this associative learning assay. This response is mediated by the AFD sensory and NSM neurons, which signal through the AIZ and AIY interneurons.

The authors modified this assay by training and testing the animals on the same plate. Scd-2 mutants showed a deficit in learning and remained on the pathogenic bacteria. fsn-1 mutants again acted similar to wild type, suggesting that fsn-1 does not act in the associative learning assay; hence, again, fsn-1 mutants cannot be used to establish epistatic relationships. Interestingly, however, the fsn-1; scd-2 double mutants acted similar to wild type, suggesting that parallel pathways are used in associative learning.

Scd-2 has different sites of action in the sensory integration and associative learning pathways. Within the sensory integration pathway, Ishihara and co-workers showed that scd-2 acts within the AIA interneurons (2011). Within the associative learning pathway, Zhang et al. (2005) showed that response to pathogenic bacteria is mediated by the ADF neurons, whereas NSM neurons mediate response to the nutritious bacteria.

The authors show that expression of scd-2 in NSM neurons rescues the associative learning defect. This result poses the question as to whether NSM neurons are slightly defective in their response to the nutritious bacteria (OP50), thereby increasing the preference of pathogenic bacteria in scd-2 mutants.

The integration of this work with previous work was not discussed in the current form of the manuscript. This integration should be provided.

SUMMARY:

The authors have determined that lrn-2 is scd-2, and scd-2 activity in the NSM neurons is required for associative learning. They should not extend their conclusions beyond these data.

MAJOR COMMENTS

1.There is a lack of consistency in the quality of writing of this manuscript. Some parts were not at all clear. The writing style feels like patchwork from different authors and some terminology is used incorrectly. For example:

--- the format of in-text reference was inconsistent: some places used authors + year, some places used a numbered reference system. These need to be corrected. What are the references 1,3,4 in line 147? What is the reference for Li et al. #16 (line 200)?

--- the format for C. elegans genes/alleles was inconsistent, some were italicized, some were not. An allele like mm99 is either lrn-2 or scd-2 in text and figures- this is confusing.

---Figures were also referred to in-text in random sequence: Some panels in Fig 1 were referred to first, then Fig 2, then the rest of the panels in Fig 1.

--- Incorrect use of the word “dissociable”. The same protein can be used in different contexts depending on developmental stage and different cellular environments. For the paradigms discussed, different neuronal circuits were examined in sensory integration and associative learning. Hence, within the different neuronal circuits, some molecules may be common to both circuits. This differential usage is not normally referred to as “dissociable”, particularly when describing learning paradigms in which dissociable has a different connotation. This terminology should be changed throughout the paper.

2. Some parts of the method were insufficiently detailed:

--- Line 80: bacteria were spotted on “opposite sides” of a 10 cm Petri dish, but how far away from the center were these two spots?

--- Line 89: the plates were incubated at which temperature?

--- Line 100: worms were raised on NGM with what food?

--- To make the protocol clearer, a schematic of the modified associative learning protocol should be included.

3. Several of the interpretations of the data/statements are not well supported and as such must be removed.

---Line 150: N2 and lrn-2 mutant both decreased egg production, this does not necessarily mean both strains can detect the pathogenicity similarly, rather, it is probably more parsimonious that biologically the PA14 is equally toxic for both strains. both strains became physically sick to similar extent, so that egg production was decreased similarly. The data thus far do not tell us anything about whether they can detect pathogenicity.

--- Fig 1B: The statement that the authors interpreted the data as N2 is able to make the association between PA14 smell and being sick, whereas scd-2 mutant is not; both strains are able to form association between PA14 smell and food value. As a result, the net behavioral response in N2 is a decrease in chemotaxis index, whereas in the mutant, it is an increase. However, it is also possible that scd-2 mutant does not know that it is sick- perhaps some metabolic feedback to tell the worms that they have been made sick by the food is not present.

--- None of the data speak to the animals ability to detect “pathogenicity”-it could be lack of awareness of illness. Do scd-2 mutants also fail to associate an odor with a non-food aversive stimulus (e.g., UV). and the experiment depicted in Fig 5A seems the opposite (copper acetate and diacetyl can be associated by scd-2 mutant).

--- Fig 5C: it looks like there is still a difference between the two groups in which copper acetate and diacetyl were not simultaneously presented. Is this difference significant? It suggests that the sequence in which copper acetate and diacetyl are presented could affect the response magnitude, thus sensory adaptation or habituation could be involved, in this case and other cases.

MINOR (but require attention)

- Line 44: What is meant by “in occasion setting”?

- Line 5 7-60: this sentence is convoluted

- Line 67: lrn-2(mm99) was repeated twice

- Line 69-70: gene and transgene names should be italicized

- Line 82: Why were L4 animals used rather than day 1 adults?

-Line 86: this is not a complete sentence

- Line 87: What stage animals were grown on OP50? - line 127: was it 30 microliters?, not 30 ml.

- Line 131-133: this sentence is hard to follow

- Line 198: down-regulation or downregulation

- Line 255 and 260: copper aversion and copper approach - I think both should be “attenuation of copper aversion”, or in the second part, just “copper approach”.

- Line 331: a typo in the last word.

- Fig 5A showed the response to copper acetate, and 5B showed the response to diacetyl, it may be better to label these so that it is clearer to the reader what the responses were in each panel.

- Fig 5C: what is the “naïve condition”? which bar in the graph corresponds to this group? It was not clearly indicated.
